# Integrating Lived Experience Into Medical Education Related to Children With Medical Complexity or Developmental Disabilities: Protocol for a Scoping Review

**DOI:** 10.2196/64911

**Published:** 2025-07-11

**Authors:** Noah Pollard, Leslie Christensen, Heidi Kloster, Danielle Gerber, Gail Chödrön

**Affiliations:** 1 University of Wisconsin School of Medicine and Public Health Madison, WI United States; 2 Ebling Library for the Health Sciences University of Wisconsin School of Medicine and Public Health Madison, WI United States; 3 Department of Pediatrics University of Wisconsin School of Medicine and Public Health Madison, WI United States; 4 Waisman Center University of Wisconsin–Madison Madison, WI United States

**Keywords:** family as educators, family as faculty, family-centered, partnership, pediatrics, medical education, family engagement in learning, children with medical complexity, children with developmental disability

## Abstract

**Background:**

Involving people with lived experience in medical education can benefit them, their caregivers, and medical students, and may particularly impact medical education about children requiring comprehensive, individualized, multidisciplinary care, including those with medical complexity or developmental disabilities. Yet, there is no summary of how children with medical complexity or developmental disabilities, or their families or caregivers have been included in medical education for medical students, residents, and fellows. To advance the effective inclusion of lived experience in medical education related to this patient population, a synthesis of existing literature is needed.

**Objective:**

This scoping review aims to identify and synthesize the literature on including the lived experiences of children with medical complexity or developmental disabilities and their caregivers in medical education, where in the curricular development process they are involved, and the level of engagement of people with lived experience in the process.

**Methods:**

To complete the scoping review, MEDLINE, Scopus, PsycINFO, ERIC, Academic Search Premier, and Google Scholar were searched for studies investigating patient and caregiver involvement in medical education related to children with medical complexity or developmental disabilities. Studies involving continuing professional development or patients who are not children with medical complexity or developmental disabilities were excluded. Data will be extracted to identify the stage of curriculum development in which lived experience is included based on Kern’s 6-step approach, and to examine the level of engagement in medical education of children with medical complexity or developmental disabilities, their families, or their caregivers. Data from the scoping review will be presented in tables, diagrams, or matrices to demonstrate how lived experience caring for children with medical complexity or children with developmental disabilities has been included in the 6 steps of curriculum development and to characterize the level of engagement of people with lived experience in this process. Descriptive analysis will be performed to identify the findings from the included sources pertaining to the research objective.

**Results:**

Database searches were completed on April 19, 2024. In total, 4382 unique articles were identified and screened against the eligibility criteria in July 2024, resulting in 30 articles being included in the scoping review. Data extraction began in July 2024.

**Conclusions:**

Our results will identify areas of improvement for medical education pertaining to the care of children with medical complexity or developmental disabilities. The findings will support and contribute to the development of medical school curricula surrounding care for children with medical complexity and children with developmental disabilities that incorporate people with lived experience in crucial roles during curriculum development. These partnerships with people with lived experience will promote more patient- and family-centered physicians, leading to better care of children with medical complexity and children with developmental disabilities.

**International Registered Report Identifier (IRRID):**

DERR1-10.2196/64911

## Introduction

### Background

The quality and outcomes of health care are impacted by providers’ knowledge and attitudes. In addition to understanding the clinical aspect of a patient’s diagnosis, a provider’s understanding of the patient and family’s lived experience may play a role in the quality of health care provided. One way by which this could be improved is through medical education. Involvement of people with lived experience, such as patients, their families, or their caregivers, in medical education is possible and can provide new insights. Furthermore, patient and caregiver experiences have already been included in multiple settings, including both educating physicians-in-training (medical students, residents, and fellows) [1-8], as well as educating practicing physicians in the setting of continuing professional development (CPD) [9-11].

Through the incorporation of people with lived experience in curriculum design for CPD, Tajani et al [9] identified experiences that would have otherwise been left out of the curriculum, shaping the curriculum objectives to have a stronger patient focus, as well as emphasizing the role patients and their families play in their care. Unique student experiences were also observed when including adult patients in medical student education. Incorporating patient experiences into medical student education has been shown to provide students with unique insights and help them challenge assumptions they held before interacting with patients and their stories [3]. Furthermore, learning directly from patients and their caregivers has been demonstrated to help students build relationships with patients and understand challenges faced by caregivers [4], with other studies finding that students involved in sessions with patients agreed that the sessions had increased their understanding of the patient experience and the application of patient- and family-centered care [6,8]. This was also observed in the pediatric setting [2], with students who interacted with patients and caregivers writing reflections that were more patient-focused than those who did not [1].

Although there is a body of literature supporting the integration of people with lived experience’s experiences in education related to adult and pediatric patients, children with medical complexity and children with developmental disabilities make up a specific subset of pediatric patients who have different experiences and care needs. Cohen et al [12] define children with medical complexity as children who have: significant health care service needs as identified by their family; at least one chronic condition that is severe or associated with fragility, whether it is diagnosed or unknown; limited function, often requiring support from technology; and high health care usage, including frequent hospitalizations, surgeries, or involvement of multiple specialists. Children with developmental disabilities have chronic impairments that are mental, physical, or both; the conditions are considered severe, must be present by the age of 22 years, are unlikely to resolve, impact multiple aspects of daily living, and lead to individuals requiring a combination of support services [13].

Due to the various needs of children with medical complexity and children with developmental disabilities, the incorporation of caregiver and patient experiences in medical curriculum development is critical. This has been demonstrated in settings of CPD [10,11], but there is a gap in understanding how it has been included in medical education for physicians in training. The methods and outcomes of involving people with lived experience in medical education must be identified to allow for effective advancement of curriculum development, specifically when designing curriculum pertaining to treating children with medical complexity and children with developmental disabilities.

As identified by previous work, the current literature on lived experiences and partnership with people with lived experience in medical education surrounding caring for children with medical complexity and children with developmental disabilities has not used consistent terms and keywords, making it difficult to identify what research and curriculum design has been completed to date [14]. A preliminary search for existing scoping reviews related to medical education involving children with medical complexity, children with developmental disabilities, and their families and caregivers was conducted in PubMed. In addition, 3 scoping reviews investigating similar education strategies were identified [15-17]; however, none of them involved children with medical complexity or children with developmental disabilities specifically. The purpose of this scoping review is to identify and synthesize literature on people with lived experience’s involvement in medical education related to children with medical complexity and children with developmental disabilities.

### Review Questions

Information known related to involving people with lived experience who are children with medical complexity or children with developmental disabilities, their families, or their caregivers in medical education is listed in Textbox 1.

**Specific questions guiding the scoping review.**

- What has been reported pertaining to the involvement of people with lived experience in medical education regarding children with medical complexity and children with developmental disability?
- What were the purposes of including people with lived experience in medical education regarding children with medical complexity and children with developmental disability?
- How were people with lived experience involved in the medical curriculum regarding children with medical complexity and children with developmental disability?
- What outcomes were measured and observed related to the impact of involving people with lived experience in medical education regarding children with medical complexity and children with developmental disability?

## Methods

### Overview

The Joanna Briggs Institute methodology for scoping reviews will be used for conducting the proposed scoping review [18]. The PRISMA-ScR (Preferred Reporting Items for Systematic reviews and Meta-Analyses extension for Scoping Reviews) will be used to report the proposed scoping review [19]. The PRISMA-P (Preferred Reporting Items for Systematic reviews and Meta-Analysis Protocols) guidelines were followed for this protocol [20]. The completed PRISMA-P checklist can be found in Multimedia Appendix 1.

### Search Strategy

The review team collaborated with a research librarian (LC) to develop and execute a comprehensive search of the literature using controlled vocabulary and title or abstract terms related to the integration of the experiences of children with medical complexity and their parents or caregivers in medical education. The search was developed in MEDLINE (EBSCO) and translated into the following databases: Scopus (Elsevier), PsycINFO (EBSCO), Education Resource Complete (EBSCO), Education Resources Information Center (EBSCO), and Academic Search Premier (EBSCO) on April 19, 2024. A Google Scholar search was performed, and the first 200 results sorted by relevance were exported for screening. No date, language, or other filters were applied to the results. The full strategy for each database has been included in Multimedia Appendix 2.

### Eligibility Criteria

The eligibility criteria were determined based on the purpose of this scoping review. Multiple criteria were identified based on the target population, concept, and context. Table 1 identifies the eligibility criteria, with further discussion of the criteria found below.

**Table 1:** Eligibility criteria.

	Include	Exclude
Population	Medical students Pediatrics residents Fellows Interprofessional education groups, including any of the above medical learners	Other health care professionals (nurses, physician assistants, etc) Practicing physicians
Concept	Patients, family, or caregivers sharing lived experiences in the curriculum Patients, family, or caregivers involved in curriculum development or design Need for patient, family, or caregiver involvement in the curriculum Patients are children with medical complexity or developmental disabilities Families are those of children with medical complexity or developmental disabilities Caregivers are those of children with medical complexity or developmental disabilities	Geriatric patients Adult patients Older patients Patients with chronic illness who are not medically complex or have developmental disabilities Families of the above patient populations Caregivers of the above patient populations
Context	Medical school Clerkship Residency Fellowship	Professional development Continuing medical education Patient education Family or caregiver education
Source type	Peer-reviewed papers	Non–peer-reviewed papers Conference proceedings Conference abstracts Grey literature Editorials Commentaries Books
Language	English	All other languages, including those translated to English
Location	N/A^a^	N/A

^a^N/A: not applicable.

### Participants

The review will include any studies involving the education of medical students, pediatric residents, or fellows in pediatric specialties. Studies investigating interprofessional education involving the previously mentioned groups will also be included. Studies focusing on other health care professionals were excluded due to the differences in roles of different professionals. Studies focused on the further education of practicing physicians were also excluded.

### Concept

This scoping review will focus on the inclusion of children with medical complexity, children with developmental disabilities, families, and caregivers in medical education curricula. Studies that relate to people with lived experiences’ involvement in curriculum development or the active participation of patients, families, or caregivers through sharing their lived experiences were included. In addition, studies investigating the need to involve patients, families, and caregivers in medical education curriculum were included. All studies involving families and caregivers that were included pertain to families and caregivers of children with medical complexity and children with developmental disabilities. Patients, families, and caregivers of pediatric patients who are not considered children with medical complexity or children with developmental disabilities were excluded, as were the lived experiences of patients, families, and caregivers of adult patients.

### Context

This scoping review will focus on medical education and curricula. This was limited to the setting of learning in medical school, clerkship, residency, and fellowship. Continuing medical education coursework and other CPD trainings were excluded. Patient, family, and caregiver education were also excluded.

### Types of Sources

The purpose of the scoping review is to gain an understanding of what information is known pertaining to people with lived experiences’ involvement in medical education surrounding children with medical complexity and children with developmental disabilities. To do this, the scoping review will include peer-reviewed experimental studies and analytical observational studies. Editorials, commentaries, abstracts, and conference proceedings were excluded.

### Evidence Screening and Selection

Results were downloaded to a citation management software (EndNote [Clarivate]) and underwent manual deduplication by LC using the method described by Bramer et al [21]. Unique records were uploaded to the Covidence (Veritas Health Innovation) screening platform for independent review by 2 independent reviewers. Disagreements between the reviewers were resolved by a third reviewer. Full text review was then completed for studies that were not excluded based on the abstract and title. This step was also completed by 2 independent reviewers, with any discrepancies being resolved by a third reviewer. The final exclusion process will be reported using a PRISMA-ScR flow diagram [19].

### Data Extraction

Data found in the papers included in the scoping review will be extracted by 2 independent reviewers using Covidence software (Veritas Health Innovation). The data extracted will include the identified need for people with lived experience involvement in medical education surrounding children with medical complexity and children with developmental disabilities, as well as how their experiences are included in medical curricula. Data involving the outcomes for medical students, residents, and fellows after taking part in these curricula will also be extracted.

A draft of the data extraction form is attached (Multimedia Appendix 3). The form will be altered throughout the extraction process as needed, with any alterations being discussed in the scoping review. Disagreements that arise between the reviewers during the data extraction process will be resolved by a third reviewer. Risk of bias will be considered by identifying papers’ funding sources and other possible conflicts of interest.

### Analysis

A total of 6 levels of people with lived experience’s engagement were defined, as adapted from frameworks created by Smits et al [22], Burns and the National Maternal and Child Health Workforce Development Center [23], McCloskey et al [24], and Shelton et al [25]. These domains were leadership, collaborative, advisory, consultative, give information, and receive information. Details about the characteristics of each level can be found in Multimedia Appendix 4. The steps of children with medical complexity and children with developmental disabilities medical curriculum development that will be mapped to are Kern’s six-step approach: (1) problem identification and general needs assessment, (2) targeted needs assessment, (3) goals and objectives, (4) educational strategies, (5) implementation, and (6) evaluation and feedback [26]. The extracted data will be analyzed using these 2 frameworks to understand people with lived experience’s engagement in curricula surrounding children with medical complexity and children with developmental disabilities, as well as the steps in curriculum development that lived experience was included.

## Results

**Figure 1:**
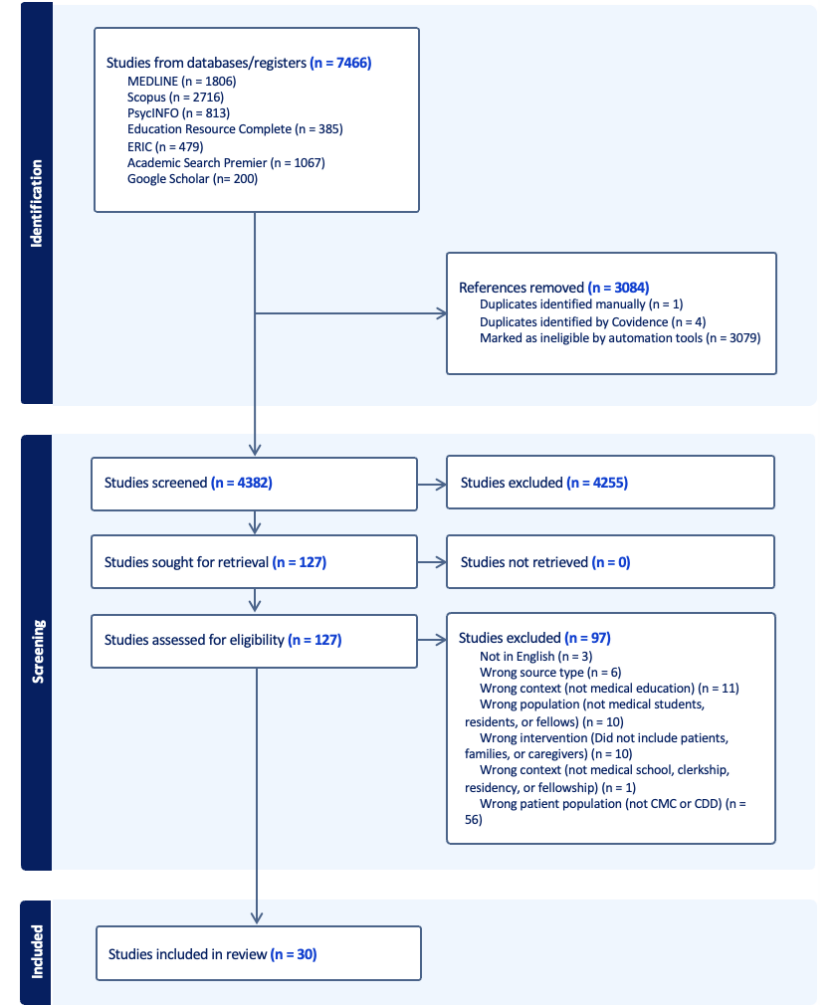
PRISMA-ScR (Preferred Reporting Items for Systematic reviews and Meta-Analyses extension for Scoping Reviews) flow diagram.

The database search was completed on April 19, 2024. A total of 7466 articles were initially identified, and duplications were removed. After deduplication, 4382 unique articles were screened. Screening began on April 19, 2024. During the screening process, 4255 articles were excluded based on the title and abstract, with an additional 97 being excluded after full text review. The screening process concluded in July of 2024. In total, 30 articles met the inclusion criteria and will undergo data extraction, with publication dates ranging from 1998 to 2023. More details regarding the screening process can be further reviewed in the PRISMA-ScR flow diagram below (Figure 1).

Upon completion of the data extraction, the extracted data will be presented in diagrams or tables to best illustrate the findings related to the review questions. The mapping of people with lived experience engagement against Kern’s steps will be presented in a matrix (Multimedia Appendix 5). In addition to the diagrams or tables and matrix, narrative descriptions of the findings will be included. The findings will be published in a scoping review.

## Discussion

### Principal Findings

There is evidence supporting the benefits of including people with lived experience in medical education, from increased student comfort in discussing sensitive topics [3] to improvement in medical students’ abilities to form relationships with patients [4]. Despite this, there is a gap in knowledge regarding people with lived experience’s involvement in medical education specific to children with medical complexity and children with developmental disabilities. This scoping review will identify the ways by which people with lived experience have been involved in medical education curriculum regarding children with medical complexity and children with developmental disabilities, what part of the curriculum they were involved in, and their level of engagement. Identifying how people with lived experience are included, such as interviews or home visits, can set the groundwork for the implementation strategies of future curricula. Identifying how people with lived experience are involved will provide insight into how early in the curriculum process people with lived experience should be involved. The levels of engagement of people with lived experience identified by this review will both highlight the importance of people with lived experience’s involvement and will also identify areas in which there is room for more engagement of people with lived experience in education surrounding the care for children with medical complexity and children with developmental disabilities. Outcomes of the curricula identified by the scoping review will highlight which of these methods are most successful in improving future physician readiness in providing quality care for children with medical complexity and children with developmental disabilities. By identifying how and when people with lived experience can best be partnered with in medical education regarding children with medical complexity and children with developmental disabilities, the authors hope that new curricula will be developed based on these findings to create educational experiences that will best prepare future physicians to provide care for this patient population. These findings will support curricular development across institutions, increasing the reach of improved training and care for children with medical complexity and children with developmental disabilities.

### Limitations

The scoping review results will be limited by the language selection, which may exclude relevant literature that is not in English. Furthermore, gray literature will be excluded, and therefore, the results may miss medical education contributions from patients, family, and caregivers. As with any scoping review, there is also the potential that there is literature surrounding this subject that was not encompassed by the keywords used for the literature search.

### Conclusions

It is the authors’ understanding that this is the first scoping review focused on the involvement of children with medical complexity, children with developmental disabilities, their families, or their caregivers in medical education. It will fill in the knowledge gaps regarding what curricula regarding care for children with medical complexity and children with developmental disabilities are currently involving people with lived experience, as well as how and when people with lived experience can be involved in curricula in ways that strengthen the learning for future physicians. This scoping review will set the groundwork for the development of future curricula that can then be disseminated across the country with the hopes of improving care for children with medical complexity and children with developmental disabilities by improving the education of their future providers.

## Data Availability

The data collected during the scoping review will be attached to the review in a multimedia appendix. The scoping review will be published and available for other researchers and educators.

## Appendix

Multimedia Appendix 1 PRISMA-P (Preferred Reporting Items for Systematic Reviews and Meta-Analysis Protocols) checklist adapted from Shamseer et al [20].

Multimedia Appendix 2 Full search strategy used for MEDLINE, Scopus, PsycINFO, ERIC, Academic Search Premier, and Google Scholar.

Multimedia Appendix 3 Draft of data extraction form used to complete the scoping review.

Multimedia Appendix 4 Definitions for the levels of engagement of people with lived experience (PLE) in medical education.

Multimedia Appendix 5 Draft of intervention matrix to map people with lived experience (PLE) engagement levels against the step in curriculum development they are involved in.

## Abbreviations

CPD: Continuing professional development
PRISMA-P: Preferred Reporting Items for Systematic Reviews and Meta-Analysis Protocols
PRISMA-ScR: Preferred Reporting Items for Systematic Reviews and Meta-Analyses extension for Scoping Reviews
