# Supplementary material for: Integrating Lived Experience Into Medical Education Related to Children With Medical Complexity or Developmental Disabilities: Protocol for a Scoping Review
Source: JMIR Res Protoc. 2025 Jul 11;14:e64911. doi: 10.2196/64911 (PMC12299940; doi:10.2196/64911)
Supplement: Multimedia Appendix 2 [file resprot_v14i1e64911_app2.docx]

**Multimedia Appendix 2: Full Search Strategy**

**EBSCO MEDLINE**Search run date: 4/19/2024
1806 Results
(((MH (“Education, Medical+” OR “Clinical Clerkship” OR “Curriculum+” OR “Students, Medical” OR “Schools, Medical” OR “Teaching”) ) OR ( TI ((medical OR medicine) N3 (educat* OR school* OR teach* OR train* OR learn* OR instruct* OR graduate* or undergrad* or postgrad* OR student*)) OR AB ((medical OR medicine) N3 (educat* OR school* OR teach* OR train* OR learn* OR instruct* OR graduate* or undergrad* or postgrad* OR student*))) OR CI ((medical OR medicine) N3 (educat* OR school* OR teach* OR train* OR learn* OR instruct* OR graduate* or undergrad* or postgrad* OR student*)) ) OR ( TI ( intern OR interns OR internee* OR internship* OR resident* OR residenc* OR fellow* OR clerkship* OR house-staff OR house-officer* OR curricul* OR competency-based ) OR AB ( intern OR interns OR internee* OR internship* OR resident* OR residenc* OR fellow* OR clerkship* OR house-staff OR house-officer* OR curricul* OR competency-based ) OR CI ( intern OR interns OR internee* OR internship* OR resident* OR residenc* OR fellow* OR clerkship* OR house-staff OR house-officer* OR curricul* OR competency-based ))) AND ((MH (“Patient-Centered Care” OR “Narrative Medicine” OR “Patient Participation”) ) OR ( TI ((patient* OR family OR families OR caregiver* OR carer* OR service-user* OR client*) N2 (centered OR centred OR partner* OR faculty OR educator* OR teacher* OR story* OR stories OR narrative* OR interview* OR discussion*)) OR AB ((patient* OR family OR families OR caregiver* OR carer* OR service-user* OR client*) N2 (centered OR centred OR partner* OR faculty OR educator* OR teacher* OR story* OR stories OR narrative* OR interview* OR discussion*)) OR CI ((patient* OR family OR families OR caregiver* OR carer* OR service-user* OR client*) N2 (centered OR centred OR partner* OR faculty OR educator* OR teacher* OR story* OR stories OR narrative* OR interview* OR discussion*)) ) OR ( TI ((person) N2 (centered OR centred)) OR AB ((person) N2 (centered OR centred)) OR CI ((person) N2 (centered OR centred)) ) OR ( TI ((patient* OR family OR families OR caregiver* OR carer* OR service-user* OR client*) N5 (participation OR perspective*)) OR AB ((patient* OR family OR families OR caregiver* OR carer* OR service-user* OR client*) N5 (participation OR perspective*)) OR CI ((patient* OR family OR families OR caregiver* OR carer* OR service-user* OR client*) N5 (participation OR perspective*)))) AND (( MH (“Disabled Children” OR “Chronic Disease” OR “Neurodevelopmental Disorders+” OR "Child Development Disorders, Pervasive+" OR "Developmental Disabilities" OR "Intellectual Disability+" OR "Abnormalities, Multiple+") ) OR ( TI (CMC OR CYSHCN) OR AB (CMC OR CYSHCN) OR CI (CMC OR CYSHCN) ) OR ( TI ((multiple) N3 (chronic OR abnormalit*)) OR AB ((multiple) N3 (chronic OR abnormalit*)) OR CI ((multiple) N3 (chronic OR abnormalit*))) OR ( TI ((medical*) N3 (complex* OR fragile*)) OR AB ((medical*) N3 (complex* OR fragile*)) OR CI ((medical*) N3 (complex* OR fragile*)) ) OR ( TI ((complex*) N2 (care OR chronic)) OR AB ((complex*) N2 (care OR chronic)) OR CI ((complex*) N2 (care OR chronic)) ) OR ( TI (handicap* OR disabled OR disabili* OR ((special) N2 (need*))) OR AB (handicap* OR disabled OR disabili* OR ((special) N2 (need*))) OR CI (handicap* OR disabled OR disabili* OR ((special) N2 (need*))) ) OR ( TI (autis* OR asperger* ) OR AB (autis* OR asperger* ) OR CI (autis* OR asperger* ) ) OR ( TI ((development*) N3 (delay* OR disorder*)) OR AB ((development*) N3 (delay* OR disorder*)) OR CI ((development*) N3 (delay* OR disorder*)) ) OR ( TI ((mental* OR intellectual*) N3 (retard* OR deficien* OR delay*)) OR AB ((mental* OR intellectual*) N3 (retard* OR deficien* OR delay*)) OR CI ((mental* OR intellectual*) N3 (retard* OR deficien* OR delay*)) ) OR ( TI ((technolog*) N2 (assist* OR dependen*)) OR AB ((technolog*) N2 (assist* OR dependen*)) OR CI ((technolog*) N2 (assist* OR dependen*)) ) OR ( TI ((chronic) N3 (disease* OR condition* OR illness*)) OR AB ((chronic) N3 (disease* OR condition* OR illness*)) OR CI ((chronic) N3 (disease* OR condition* OR illness*)))))

---

**Scopus**Search run date: 4/19/2024
2716 Results
(TITLE-ABS-KEY(((medical OR medicine) W/3 (educat* OR school* OR teach* OR train* OR learn* OR instruct* OR graduate* or undergrad* or postgrad* OR student*)) OR intern OR interns OR internee* OR internship* OR resident* OR residenc* OR fellow* OR clerkship* OR house-staff OR house-officer* OR curricul* OR competency-based)) AND (TITLE-ABS-KEY(((patient* OR family OR families OR caregiver* OR carer* OR service-user* OR client*) W/2 (centered OR centred OR partner* OR faculty OR educator* OR teacher* OR story* OR stories OR narrative* OR interview* OR discussion*)) OR ((person) W/2 (centered OR centred)) OR ((patient* OR family OR families OR caregiver* OR carer* OR service-user* OR client*) W/5 (participation OR perspective*)))) AND (TITLE-ABS-KEY(CMC OR CYSHCN OR ((multiple) W/3 (chronic OR abnormalit*)) OR ((medical*) W/3 (complex* OR fragile*)) OR ((complex*) W/2 (care OR chronic)) OR handicap* OR disabled OR disabili* OR ((special) W/2 (need*)) OR autis* OR asperger* OR ((development*) W/3 (delay* OR disorder*)) OR ((mental* OR intellectual*) W/3 (retard* OR deficien* OR delay*)) OR ((technolog*) W/2 (assist* OR dependen*)) OR ((chronic) W/3 (disease* OR condition* OR illness*))))

**PsycINFO**Search run date: 4/19/2024
813 Results
(((DE (“Medical Education” OR “Medical Internship” OR “Medical Residency” OR “Curriculum” OR “Medical Students” OR “Teaching”) ) OR ( TI ((medical OR medicine) N3 (educat* OR school* OR teach* OR train* OR learn* OR instruct* OR graduate* or undergrad* or postgrad* OR student*)) OR AB ((medical OR medicine) N3 (educat* OR school* OR teach* OR train* OR learn* OR instruct* OR graduate* or undergrad* or postgrad* OR student*))) ) OR ( TI ( intern OR interns OR internee* OR internship* OR resident* OR residenc* OR fellow* OR clerkship* OR house-staff OR house-officer* OR curricul* OR competency-based ) OR AB ( intern OR interns OR internee* OR internship* OR resident* OR residenc* OR fellow* OR clerkship* OR house-staff OR house-officer* OR curricul* OR competency-based ))) AND ((DE (“Patient Centered Care” OR “Storytelling” OR “Narratives” OR “Client Participation”) ) OR ( TI ((patient* OR family OR families OR caregiver* OR carer* OR service-user* OR client*) N2 (centered OR centred OR partner* OR faculty OR educator* OR teacher* OR story* OR stories OR narrative* OR interview* OR discussion*)) OR AB ((patient* OR family OR families OR caregiver* OR carer* OR service-user* OR client*) N2 (centered OR centred OR partner* OR faculty OR educator* OR teacher* OR story* OR stories OR narrative* OR interview* OR discussion*))) OR ( TI ((person) N2 (centered OR centred)) OR AB ((person) N2 (centered OR centred))) OR ( TI ((patient* OR family OR families OR caregiver* OR carer* OR service-user* OR client*) N5 (participation OR perspective*)) OR AB ((patient* OR family OR families OR caregiver* OR carer* OR service-user* OR client*) N5 (participation OR perspective*)))) AND (( DE ( “Chronic Illness” OR “Chronically Ill Children” OR "Neurodevelopmental Disorders" OR "Autism Spectrum Disorders" OR "Developmental Disabilities" OR "Intellectual Development Disorder" OR "Multiple Disabilities” ) OR ( TI (CMC OR CYSHCN) OR AB (CMC OR CYSHCN)) OR ( TI ((multiple) N3 (chronic OR abnormalit*)) OR AB ((multiple) N3 (chronic OR abnormalit*))) OR ( TI ((medical*) N3 (complex* OR fragile*)) OR AB ((medical*) N3 (complex* OR fragile*)) OR CI ((medical*) N3 (complex* OR fragile*)) ) OR ( TI ((complex*) N2 (care OR chronic)) OR AB ((complex*) N2 (care OR chronic))) OR ( TI (handicap* OR disabled OR disabili* OR ((special) N2 (need*))) OR AB (handicap* OR disabled OR disabili* OR ((special) N2 (need*))) ) OR ( TI (autis* OR asperger* ) OR AB (autis* OR asperger* ) ) OR ( TI ((development*) N3 (delay* OR disorder*)) OR AB ((development*) N3 (delay* OR disorder*)) OR ) OR ( TI ((mental* OR intellectual*) N3 (retard* OR deficien* OR delay*)) OR AB ((mental* OR intellectual*) N3 (retard* OR deficien* OR delay*))) OR ( TI ((technolog*) N2 (assist* OR dependen*)) OR AB ((technolog*) N2 (assist* OR dependen*))) OR ( TI ((chronic) N3 (disease* OR condition* OR illness*)) OR AB ((chronic) N3 (disease* OR condition* OR illness*)))))

---

**Education Resource Complete**
Search run date: 4/19/2024
385 Results
((( TI ((medical OR medicine) N3 (educat* OR school* OR teach* OR train* OR learn* OR instruct* OR graduate* or undergrad* or postgrad* OR student*)) OR AB ((medical OR medicine) N3 (educat* OR school* OR teach* OR train* OR learn* OR instruct* OR graduate* or undergrad* or postgrad* OR student*))) OR SU ((medical OR medicine) N3 (educat* OR school* OR teach* OR train* OR learn* OR instruct* OR graduate* or undergrad* or postgrad* OR student*)) ) OR ( TI ( intern OR interns OR internee* OR internship* OR resident* OR residenc* OR fellow* OR clerkship* OR house-staff OR house-officer* OR curricul* OR competency-based ) OR AB ( intern OR interns OR internee* OR internship* OR resident* OR residenc* OR fellow* OR clerkship* OR house-staff OR house-officer* OR curricul* OR competency-based ) OR SU ( intern OR interns OR internee* OR internship* OR resident* OR residenc* OR fellow* OR clerkship* OR house-staff OR house-officer* OR curricul* OR competency-based ))) AND (( TI ((patient* OR family OR families OR caregiver* OR carer* OR service-user* OR client*) N2 (centered OR centred OR partner* OR faculty OR educator* OR teacher* OR story* OR stories OR narrative* OR interview* OR discussion*)) OR AB ((patient* OR family OR families OR caregiver* OR carer* OR service-user* OR client*) N2 (centered OR centred OR partner* OR faculty OR educator* OR teacher* OR story* OR stories OR narrative* OR interview* OR discussion*)) OR SU ((patient* OR family OR families OR caregiver* OR carer* OR service-user* OR client*) N2 (centered OR centred OR partner* OR faculty OR educator* OR teacher* OR story* OR stories OR narrative* OR interview* OR discussion*)) ) OR ( TI ((person) N2 (centered OR centred)) OR AB ((person) N2 (centered OR centred)) OR SU ((person) N2 (centered OR centred)) ) OR ( TI ((patient* OR family OR families OR caregiver* OR carer* OR service-user* OR client*) N5 (participation OR perspective*)) OR AB ((patient* OR family OR families OR caregiver* OR carer* OR service-user* OR client*) N5 (participation OR perspective*)) OR SU ((patient* OR family OR families OR caregiver* OR carer* OR service-user* OR client*) N5 (participation OR perspective*)))) AND (( TI (CMC OR CYSHCN) OR AB (CMC OR CYSHCN) OR SU (CMC OR CYSHCN) ) OR ( TI ((multiple) N3 (chronic OR abnormalit*)) OR AB ((multiple) N3 (chronic OR abnormalit*)) OR SU ((multiple) N3 (chronic OR abnormalit*))) OR ( TI ((medical*) N3 (complex* OR fragile*)) OR AB ((medical*) N3 (complex* OR fragile*)) OR SU ((medical*) N3 (complex* OR fragile*)) ) OR ( TI ((complex*) N2 (care OR chronic)) OR AB ((complex*) N2 (care OR chronic)) OR SU ((complex*) N2 (care OR chronic)) ) OR ( TI (handicap* OR disabled OR disabili* OR ((special) N2 (need*))) OR AB (handicap* OR disabled OR disabili* OR ((special) N2 (need*))) OR SU (handicap* OR disabled OR disabili* OR ((special) N2 (need*))) ) OR ( TI (autis* OR asperger* ) OR AB (autis* OR asperger* ) OR SU (autis* OR asperger* ) ) OR ( TI ((development*) N3 (delay* OR disorder*)) OR AB ((development*) N3 (delay* OR disorder*)) OR SU ((development*) N3 (delay* OR disorder*)) ) OR ( TI ((mental* OR intellectual*) N3 (retard* OR deficien* OR delay*)) OR AB ((mental* OR intellectual*) N3 (retard* OR deficien* OR delay*)) OR SU ((mental* OR intellectual*) N3 (retard* OR deficien* OR delay*)) ) OR ( TI ((technolog*) N2 (assist* OR dependen*)) OR AB ((technolog*) N2 (assist* OR dependen*)) OR SU ((technolog*) N2 (assist* OR dependen*)) ) OR ( TI ((chronic) N3 (disease* OR condition* OR illness*)) OR AB ((chronic) N3 (disease* OR condition* OR illness*)) OR SU ((chronic) N3 (disease* OR condition* OR illness*)))))

---

**ERIC**Search run date: 4/19/2024
479 Results
((( TI ((medical OR medicine) N3 (educat* OR school* OR teach* OR train* OR learn* OR instruct* OR graduate* or undergrad* or postgrad* OR student*)) OR AB ((medical OR medicine) N3 (educat* OR school* OR teach* OR train* OR learn* OR instruct* OR graduate* or undergrad* or postgrad* OR student*))) OR SU ((medical OR medicine) N3 (educat* OR school* OR teach* OR train* OR learn* OR instruct* OR graduate* or undergrad* or postgrad* OR student*)) ) OR ( TI ( intern OR interns OR internee* OR internship* OR resident* OR residenc* OR fellow* OR clerkship* OR house-staff OR house-officer* OR curricul* OR competency-based ) OR AB ( intern OR interns OR internee* OR internship* OR resident* OR residenc* OR fellow* OR clerkship* OR house-staff OR house-officer* OR curricul* OR competency-based ) OR SU ( intern OR interns OR internee* OR internship* OR resident* OR residenc* OR fellow* OR clerkship* OR house-staff OR house-officer* OR curricul* OR competency-based ))) AND (( TI ((patient* OR family OR families OR caregiver* OR carer* OR service-user* OR client*) N2 (centered OR centred OR partner* OR faculty OR educator* OR teacher* OR story* OR stories OR narrative* OR interview* OR discussion*)) OR AB ((patient* OR family OR families OR caregiver* OR carer* OR service-user* OR client*) N2 (centered OR centred OR partner* OR faculty OR educator* OR teacher* OR story* OR stories OR narrative* OR interview* OR discussion*)) OR SU ((patient* OR family OR families OR caregiver* OR carer* OR service-user* OR client*) N2 (centered OR centred OR partner* OR faculty OR educator* OR teacher* OR story* OR stories OR narrative* OR interview* OR discussion*)) ) OR ( TI ((person) N2 (centered OR centred)) OR AB ((person) N2 (centered OR centred)) OR SU ((person) N2 (centered OR centred)) ) OR ( TI ((patient* OR family OR families OR caregiver* OR carer* OR service-user* OR client*) N5 (participation OR perspective*)) OR AB ((patient* OR family OR families OR caregiver* OR carer* OR service-user* OR client*) N5 (participation OR perspective*)) OR SU ((patient* OR family OR families OR caregiver* OR carer* OR service-user* OR client*) N5 (participation OR perspective*)))) AND (( TI (CMC OR CYSHCN) OR AB (CMC OR CYSHCN) OR SU (CMC OR CYSHCN) ) OR ( TI ((multiple) N3 (chronic OR abnormalit*)) OR AB ((multiple) N3 (chronic OR abnormalit*)) OR SU ((multiple) N3 (chronic OR abnormalit*))) OR ( TI ((medical*) N3 (complex* OR fragile*)) OR AB ((medical*) N3 (complex* OR fragile*)) OR SU ((medical*) N3 (complex* OR fragile*)) ) OR ( TI ((complex*) N2 (care OR chronic)) OR AB ((complex*) N2 (care OR chronic)) OR SU ((complex*) N2 (care OR chronic)) ) OR ( TI (handicap* OR disabled OR disabili* OR ((special) N2 (need*))) OR AB (handicap* OR disabled OR disabili* OR ((special) N2 (need*))) OR SU (handicap* OR disabled OR disabili* OR ((special) N2 (need*))) ) OR ( TI (autis* OR asperger* ) OR AB (autis* OR asperger* ) OR SU (autis* OR asperger* ) ) OR ( TI ((development*) N3 (delay* OR disorder*)) OR AB ((development*) N3 (delay* OR disorder*)) OR SU ((development*) N3 (delay* OR disorder*)) ) OR ( TI ((mental* OR intellectual*) N3 (retard* OR deficien* OR delay*)) OR AB ((mental* OR intellectual*) N3 (retard* OR deficien* OR delay*)) OR SU ((mental* OR intellectual*) N3 (retard* OR deficien* OR delay*)) ) OR ( TI ((technolog*) N2 (assist* OR dependen*)) OR AB ((technolog*) N2 (assist* OR dependen*)) OR SU ((technolog*) N2 (assist* OR dependen*)) ) OR ( TI ((chronic) N3 (disease* OR condition* OR illness*)) OR AB ((chronic) N3 (disease* OR condition* OR illness*)) OR SU ((chronic) N3 (disease* OR condition* OR illness*)))))

---

**Academic Search Premier**Search run date: 4/19/2024
1,067 Results

((( TI ((medical OR medicine) N3 (educat* OR school* OR teach* OR train* OR learn* OR instruct* OR graduate* or undergrad* or postgrad* OR student*)) OR AB ((medical OR medicine) N3 (educat* OR school* OR teach* OR train* OR learn* OR instruct* OR graduate* or undergrad* or postgrad* OR student*))) OR SU ((medical OR medicine) N3 (educat* OR school* OR teach* OR train* OR learn* OR instruct* OR graduate* or undergrad* or postgrad* OR student*)) ) OR ( TI ( intern OR interns OR internee* OR internship* OR resident* OR residenc* OR fellow* OR clerkship* OR house-staff OR house-officer* OR curricul* OR competency-based ) OR AB ( intern OR interns OR internee* OR internship* OR resident* OR residenc* OR fellow* OR clerkship* OR house-staff OR house-officer* OR curricul* OR competency-based ) OR SU ( intern OR interns OR internee* OR internship* OR resident* OR residenc* OR fellow* OR clerkship* OR house-staff OR house-officer* OR curricul* OR competency-based ))) AND (( TI ((patient* OR family OR families OR caregiver* OR carer* OR service-user* OR client*) N2 (centered OR centred OR partner* OR faculty OR educator* OR teacher* OR story* OR stories OR narrative* OR interview* OR discussion*)) OR AB ((patient* OR family OR families OR caregiver* OR carer* OR service-user* OR client*) N2 (centered OR centred OR partner* OR faculty OR educator* OR teacher* OR story* OR stories OR narrative* OR interview* OR discussion*)) OR SU ((patient* OR family OR families OR caregiver* OR carer* OR service-user* OR client*) N2 (centered OR centred OR partner* OR faculty OR educator* OR teacher* OR story* OR stories OR narrative* OR interview* OR discussion*)) ) OR ( TI ((person) N2 (centered OR centred)) OR AB ((person) N2 (centered OR centred)) OR SU ((person) N2 (centered OR centred)) ) OR ( TI ((patient* OR family OR families OR caregiver* OR carer* OR service-user* OR client*) N5 (participation OR perspective*)) OR AB ((patient* OR family OR families OR caregiver* OR carer* OR service-user* OR client*) N5 (participation OR perspective*)) OR SU ((patient* OR family OR families OR caregiver* OR carer* OR service-user* OR client*) N5 (participation OR perspective*)))) AND (( TI (CMC OR CYSHCN) OR AB (CMC OR CYSHCN) OR SU (CMC OR CYSHCN) ) OR ( TI ((multiple) N3 (chronic OR abnormalit*)) OR AB ((multiple) N3 (chronic OR abnormalit*)) OR SU ((multiple) N3 (chronic OR abnormalit*))) OR ( TI ((medical*) N3 (complex* OR fragile*)) OR AB ((medical*) N3 (complex* OR fragile*)) OR SU ((medical*) N3 (complex* OR fragile*)) ) OR ( TI ((complex*) N2 (care OR chronic)) OR AB ((complex*) N2 (care OR chronic)) OR SU ((complex*) N2 (care OR chronic)) ) OR ( TI (handicap* OR disabled OR disabili* OR ((special) N2 (need*))) OR AB (handicap* OR disabled OR disabili* OR ((special) N2 (need*))) OR SU (handicap* OR disabled OR disabili* OR ((special) N2 (need*))) ) OR ( TI (autis* OR asperger* ) OR AB (autis* OR asperger* ) OR SU (autis* OR asperger* ) ) OR ( TI ((development*) N3 (delay* OR disorder*)) OR AB ((development*) N3 (delay* OR disorder*)) OR SU ((development*) N3 (delay* OR disorder*)) ) OR ( TI ((mental* OR intellectual*) N3 (retard* OR deficien* OR delay*)) OR AB ((mental* OR intellectual*) N3 (retard* OR deficien* OR delay*)) OR SU ((mental* OR intellectual*) N3 (retard* OR deficien* OR delay*)) ) OR ( TI ((technolog*) N2 (assist* OR dependen*)) OR AB ((technolog*) N2 (assist* OR dependen*)) OR SU ((technolog*) N2 (assist* OR dependen*)) ) OR ( TI ((chronic) N3 (disease* OR condition* OR illness*)) OR AB ((chronic) N3 (disease* OR condition* OR illness*)) OR SU ((chronic) N3 (disease* OR condition* OR illness*)))))

---

**Google Scholar**Search run date: 4/19/2024
200 results
"Medical education"|"medical school"|"medical schools"|"medical student"|"medical students"|residency|resident|residents|curriculum|curricula “medical complexity”|”medical complexities"|"complex care" families|caregiver|caregivers|carer|carers|patient|patients partner|partnering|partnership|partnerships|”co develop”|participate|participates
