# Supplementary material for: Integrating Lived Experience Into Medical Education Related to Children With Medical Complexity or Developmental Disabilities: Protocol for a Scoping Review
Source: JMIR Res Protoc. 2025 Jul 11;14:e64911. doi: 10.2196/64911 (PMC12299940; doi:10.2196/64911)
Supplement: Multimedia Appendix 3 [file resprot_v14i1e64911_app3.docx]

**Multimedia Appendix 3: Draft of Data Extraction Form**

| Study Identification | | | |
| --- | --- | --- | --- |
|  | Consensus | Reviewer 1 | Reviewer 2 |
| Article Title |  |  |  |
| Journal/Book/Magazine Title |  |  |  |
| Year of Publication |  |  |  |
| Last Name of First Author |  |  |  |
| Institution |  |  |  |
| Country |  |  |  |
| Methods | | | |
|  | Consensus | Reviewer 1 | Reviewer 2 |
| Study Design |  |  |  |
| Methodology |  |  |  |
| Aim of Study |  |  |  |
| Setting |  |  |  |
| Purpose for Lived Experience Inclusion |  |  |  |
| Population | | | |
|  | Consensus | Reviewer 1 | Reviewer 2 |
| Learner Level (pre-clinical medical students, clinical medical students, residents, fellows) |  |  |  |
| Number of Learners |  |  |  |
| Person with lived experience (patients, parents/caregivers, both) |  |  |  |
| Number of Students per PLE |  |  |  |
| Other Information About PLE |  |  |  |
| Interventions | | | |
|  | Consensus | Reviewer 1 | Reviewer 2 |
| What Learners Experienced (home visit, lecture, video, etc.) |  |  |  |
| Step of Curriculum Development  (Kern’s Step) |  |  |  |
| Level of Engagement (Leadership, Collaborative, Advisory, Consultative, Give Information, Receive Information) |  |  |  |
| Results | | | |
|  | Consensus | Reviewer 1 | Reviewer 2 |
| Validated tool used (Y/N)? |  |  |  |
| If yes, what tool? |  |  |  |
| Learners Evaluated (Y/N)? |  |  |  |
| If yes, results? |  |  |  |
| Families Evaluated (Y/N)? |  |  |  |
| If yes, results? |  |  |  |
| Outcomes | | | |
|  | Consensus | Reviewer 1 | Reviewer 2 |
| Author’s Conclusions |  |  |  |
| Reviewer’s Comments |  |  |  |
| Potential Bias | | | |
|  | Consensus | Reviewer 1 | Reviewer 2 |
| Funding Sources |  |  |  |
| Possible Conflicts of Interest |  |  |  |
