# Supplementary material for: Integrating Lived Experience Into Medical Education Related to Children With Medical Complexity or Developmental Disabilities: Protocol for a Scoping Review
Source: JMIR Res Protoc. 2025 Jul 11;14:e64911. doi: 10.2196/64911 (PMC12299940; doi:10.2196/64911)
Supplement: Multimedia Appendix 4 [file resprot_v14i1e64911_app4.docx]

**Multimedia Appendix 4: PLE Levels of Engagement**

| **Engagement Level** | **Characteristics of Level of Engagement** |
| --- | --- |
| **Leadership** | PLE serve in leadership roles and share in decision-making. |
| **Collaborative** | PLE serve as ongoing collaborators/partners in the work in ways that shape project priorities, aims, approach, and/or strategies used. |
| **Advisory** | PLE fill recognized, ongoing advisory roles. The PLE advisory relationship may be limited to project leadership rather than full project team. PLE have some influence on, but are not the decision-makers about, how and when their input is used. |
| **Consultative** | PLE are consulted on a sporadic basis using bidirectional communication strategies. PLE have little to no influence on how and when their input is used. |
| **Give Information** | PLE provide information on a one-time or sporadic basis through surveys, interviews, storytelling, and other methods. PLE are not involved in shaping how and when the information is used. |
| **Receive Information** | Project information is disseminated to PLE. |
