# Supplementary material for: Integrating Lived Experience Into Medical Education Related to Children With Medical Complexity or Developmental Disabilities: Protocol for a Scoping Review
Source: JMIR Res Protoc. 2025 Jul 11;14:e64911. doi: 10.2196/64911 (PMC12299940; doi:10.2196/64911)
Supplement: Multimedia Appendix 5 [file resprot_v14i1e64911_app5.docx]

**Draft of Intervention Matrix**

|  | | **Kern’s Steps** | | | | | |
| --- | --- | --- | --- | --- | --- | --- | --- |
|  |  | **Step 1** | **Step 2** | **Step 3** | **Step 4** | **Step 5** | **Step 6** |
| **Level of Engagement** | **Leadership** |  |  |  |  |  |  |
|  | **Collaborative** |  |  |  |  |  |  |
|  | **Advisory** |  |  |  |  |  |  |
|  | **Consultative** |  |  |  |  |  |  |
|  | **Give Information** |  |  |  |  |  |  |
|  | **Receive Information** |  |  |  |  |  |  |
